# Evaluating the Impact of Carbon Nanoparticles on the Interfacial Properties of the Pulmonary Surfactant Film

**DOI:** 10.3390/nano15161244

**Published:** 2025-08-14

**Authors:** Yingxue Geng, Qun Zhao, Junfeng Wang, Yan Cao, Yunshan Wang, Wenshi Gou, Linfeng Zhang, Senlin Tian

**Affiliations:** 1 Faculty of Civil and Hydraulic Engineering, Xichang University, Xichang 615013, China; xcc04100056@xcc.edu.cn (Y.G.); xcc04100057@xcc.edu.cn (J.W.); xcc03000038@xcc.edu.cn (Y.W.); xcxygws@xcc.edu.cn (W.G.); 2 Faculty of Environmental Science and Engineering, Kunming University of Science and Technology, Kunming 650500, China; 20250072@kust.edu.cn (Y.C.); zhanglinfeng@stu.kust.edu.cn (L.Z.)

**Keywords:** carbon nanoparticles, pulmonary surfactant, interfacial properties, relaxation mechanisms, adsorption

## Abstract

The interaction between carbon nanoparticles (CNs) and Langmuir monolayers of 1,2-dipalmitoyl-sn-glycero-3-phosphocholine (DPPC) as a model pulmonary surfactant (PS) film was studied to shed light on the physicochemical bases underlying the potential adverse effects associated with pollutant inhalation. The results indicated that the surface pressure–area isotherms of the DPPC monolayers shifted toward lower molecular areas, and the compression modulus was reduced in the presence of CNs, hindering the ability of the DPPC monolayers to reduce the surface tension. The relaxation process of the DPPC monolayers were influenced, and the surface morphology and the continuity of the monolayers were destroyed by the penetration of CNs into the DPPC monolayers. The molecular dynamics simulation revealed that particle incorporation into the DPPC monolayers reduced the packing density of the DPPC molecules, worsening the mechanical performance of the monolayers. This effect was attributed to the strong binding trend between the CNs and the DPPC molecules. These results demonstrated that CNs could alter the relaxation mechanisms of the PS film, and this may cause a modification of the inhaled particle transport at the PS film and contribute to adverse health effects in the respiratory system of workers involved in the CN production process.

## 1. Introduction

Carbon nanoparticles (CNs), such as graphene, fullerene, and carbon nanotubes, are widely produced and used in a large array of fields [1,2]. The widespread use of CNs has raised many questions regarding the potential risks associated with their inhalation. Evidence has shown that CNs can lead to respiratory, circulatory, immune, and nervous system diseases, and they are especially highly related to pneumonia, lung fibrosis, and chronic obstructive lung disease [3,4,5]. The reason that CNs produced toxicity is that they can penetrate a range of biological barriers within the human body, such as the skin, the alveolar–capillary barrier, the blood–brain barrier, and the blood–placental barrier [6,7]; hence, they reach all parts of life [8,9]. It is estimated that the surface area of lung tissue exposed to the environment is as high as 120 m^2^, and this is where effective gas exchange occurs during breathing. CNs can deposit in the alveoli lining through inhalation and directly interact with the pulmonary surfactant (PS) film [10,11]. It is clear that the PS film has the primary functions of regulating the alveoli surface tension, increasing lung compliance, and stabilizing the alveolar volume. The PS film is one of the first biological barriers against inhaled pollutants and respiratory pathogens (e.g., SARS-CoV-2). Thus, understanding the interaction of CNs with the PS film is particularly important and may be useful as a preliminary tool to reveal the potential toxicity of CNs.

Inhaled particles, regardless of the nature of their surfaces, are submersed into the lining layer after deposition in small airways and alveoli. Many studies have focused on the evaluation of CN cytotoxicity and targeted drug delivery. It is important to note that Nemmar et al. [12] showed the passage of inhaled CNs into the blood circulation in humans. Guzman et al. [13] reported a drop in the bending rigidity of lipid layers following the insertion of CNs into lipid films. The observed adverse effects of CNs have been further explained based on the oxidative stress paradigm [14]. Molecular dynamic simulations have revealed that CNs can induce stable pores in the lipid bilayer, affecting the structures and mechanical properties of the lipid bilayer as well as leading to dynamic interactions [15]. These studies exploited many unique properties of CNs, such as the particle size, shape, hydrophilicity, and hydrophobicity, to evaluate the lung toxicity effects of CNs. However, the current understanding of the potential biophysical modifications of the PS film associated with CNs remains far from clear. On the one hand, the interaction of CNs with the PS film alters the interfacial properties, whereas on the other hand, the use of CNs is widespread in therapy and diagnostic methods. Many studies can be found in the literature regarding PS film interactions with various carbon-based nanoparticles. Most of these studies have focused on the modification induced by the incorporation of particles on the surface pressure of the interface [16,17,18]. Guzman et al. studied the effect of the incorporation of CNs into DPPC monolayers on the basis of the modification of the equilibrium isotherm and rheological properties of DPPC. They found that the impoverishment of the lipid composition of the interface is inferred to be due to lipid adsorption onto the CN surface. However, the adsorption effect of particles on phospholipid monolayers has not been directly confirmed. Although these studies indicated an association between exposure to CNs and a reduced function of the PS film, the specific effect mechanism requires further investigation, especially for the phase behavior and surface activity of the PS film. Moreover, less attention has been paid to a comparison of the adverse effects of several CNs on the interfacial properties of the PS film.

Interfacial sensitive techniques have emerged as a very promising approach for evaluating the effect of different particles on the PS film in vitro. The most extended model used in studies related to the interaction of the PS film with nanoparticles is 1,2-dipalmitoyl-sn-glycero-3-phosphocholine (DPPC), which is the primary component of the PS film (approximately 40 wt.% of the total weight), playing a key role in the ability of the PS film to decrease the surface tension to quasi-null values [19,20]. DPPC monolayers can be considered a good tool for a preliminary model construction of the PS film to evaluate the worsening of the physicochemical properties of the PS film as a result of the incorporation of CNs. Although the chemical composition of the DPPC monolayers is simpler than the natural PS film, the use of DPPC monolayers as a model system has been experimentally demonstrated to simulate certain biophysical properties of the natural PS film, such as the dynamic surface activity, biomechanics, and phospholipid phase separation upon film compression.

This study explores the interaction between CNs and the PS film using in vitro experimental assays based on the changes in the interfacial properties of the DPPC monolayers. Nanocarbon powder (NCP), graphene oxide (GO), and carbon nanotubes (CNTs) were selected as the targets to study their impact in the interfacial rheology properties of the DPPC monolayers, including the surface pressure–area isotherms, the elastic modulus, the relaxation isotherms, the Brewster angle microscope (BAM), and the atomic force microscope (AFM). Furthermore, other structural and dynamic properties of the DPPC monolayers induced by the incorporation of CNs were studied using a molecular dynamics (MD) simulation to reveal the interaction mechanism at the molecular level. The CNs-DPPC interaction, the variation in the phase behavior, and the microstructure of the DPPC monolayers disturbed by CNs can provide evidence of the inhibitory effect of CNs on the PS film and even explain the adverse effect of CNs on the compliance of lung ventilation.

## 2. Materials and Methods

### 2.1. Materials

The DPPC (purity > 99%) was obtained from Sigma-Aldrich (St. Louis, MO, USA). The nanocarbon powder (NCP) (diameter: 30 nm, purity > 99.5%) was purchased from the Macklin Biochemical Co., Ltd. (Shanghai, China). The graphene oxide (GO) powder with a nominal purity of >99% was provided by the XFNANO Materials Tech. Co., Ltd. (Nanjing, China), and the carbon nanotubes (CNTs) (diameter: 20–40 nm, length < 2 µm, purity > 97%) were purchased from the Nanoport Co., Ltd. (Shenzhen, China). The basic properties of the CNs are provided in Table S1. The micromorphology, size distribution, and zeta-potentials of the CNs in pure water are presented in Figure S1, Figure S2, and Figure S3, respectively. The hydrophilicity/hydrophobicity of the CNs are shown in Text S1 and Figure S4. Milli-Q purified water with a resistivity of 18.25 MΩ·cm was utilized for all of the experiments, and all experiments were prepared by dissolving the ingredients in saline solution (0.9% NaCl). The pH of subphase was adjusted to 7.0 ± 0.2 using HCl/NaOH.

### 2.2. Effect of CNS on the Surface Pressure–Area (π-A Isotherms) of the DPPC Monolayers

The π-A isotherms were obtained using the multifunctional Langmuir Wilhelmy (L-W) film balance (JML04C2, Zhongchen Digital Technology Equipment Co., Ltd., Shanghai, China). The DPPC was dissolved in chloroform at a concentration of 1.0 mM. The CNs were diluted with a saline solution to different concentrations as the subphase. A total of 260 mL of the subphase solution was poured into a tank, and the DPPC/chloroform solution was spread on the air–water interface by dropwise addition using a Hamilton microsyringe. A wait time of 20 min was used to ensure the complete evaporation of the chloroform, then the sliding barrier was controlled to compress the DPPC monolayers evenly at a rate of 15.5 mm/min, and the corresponding π-A isotherms were recorded. The temperature of the subphase was maintained at a constant of (37 ± 0.5) °C using a circulating water system.

### 2.3. Surface Pressure–Time (π-t) Curves

As described in the π-A isotherms experiment, the CN subphase solution was poured into a tank, and the DPPC/chloroform solution was spread on the air–water interface by dropwise addition using a Hamilton microsyringe. The device was set to the π-t mode. The barriers were then compressed to the proper position (π = 30 mN/m), and the change in the surface pressure as a function of time (i.e., π-t curves) was recorded automatically.

### 2.4. Characterization of the Langmuir–Blodgett (LB) Monolayers Using BAM

A total of 60 mL of the CN solution at a concentration of 10 mg/L was poured into the sample tank, and the DPPC/chloroform solution was spread over the air–water interface by dropwise addition using a Hamilton microsyringe. The chloroform was left until evaporation, and the DPPC monolayers were then compressed to 30 mN/m. BAM (Nanofilm-EP4 BAM, Accurion, Germany) was employed to scan the micromorphology of the flowing DPPC monolayers.

### 2.5. LB Monolayers Characterized Using AFM

The preparation of the LB monolayers was performed in the L-W film balance. The DPPC monolayers were transferred to a mica plate using the vertical lifting method (Z-type) at a speed of 2.25 mm/min and at a constant surface pressure of 30 mN/m. AFM (Agilent 5500, Agilent Technologies Co. Ltd., Santa Clara, CA, USA) was employed to scan the surface topography of the DPPC monolayers in tapping mode with a scanning area of 10 × 10 μm.

### 2.6. Computational Details

**Figure 1:**
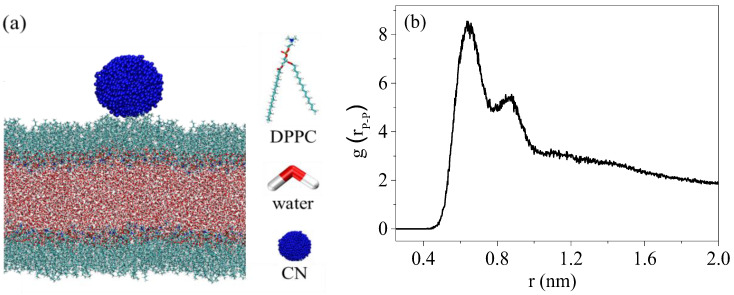
Construction of the DPPC monolayers and CN model (**a**); the radial distribution of P-P atoms in the DPPC monolayers (**b**).

Atomistic molecular dynamics simulations were performed in the GROMACS (version 2020.6) simulation package using the CHARMM 36 force field [21,22]. A bi-monolayer system was used to probe the interactions between the CNs and the DPPC monolayers. The monolayers of 200 DPPC systems were constructed on both sides of a water slab modeled using the TIP3P water model (Figure 1a). DPPC plane was set parallel to the XY plane of the system, and DPPC molecules were oriented with the polar heads directed towards to the water phase. Periodic boundary in XY direction. The simulation was performed in the NVT ensemble (constant particle number, volume, and temperature).

A spherical nanoparticle of 3.0 nm amorphous carbon was constructed using the Build Nanocluster module of the materials studio simulation software (Figure S5). We used the x2top command to generate an NP structure file. The bond length and bond angular potential functions and parameters of the CNs are as follows:

(1) $$V_{\mathrm{bonded}}=\sum_{\mathrm{bonds}}\frac{1}{2}k_{b}{\left(l-l_{0}\right)}^{2}+\sum_{\mathrm{angles}}\frac{1}{2}{k_{\theta }\left(\theta -\theta_{0}\right)}^{2}$$

(2) $$V_{\mathrm{nonbonded}}=\sum_{i=1}^{N}\sum_{j=i+1}^{N}\left\{\frac{q_{i}q_{i}}{4\pi \epsilon_{0}\epsilon_{r}r_{ij}}+4\varepsilon_{ij}\left[{\left(\frac{\sigma_{ij}}{r_{ij}}\right)}^{12}-{\left(\frac{\sigma_{ij}}{r_{ij}}\right)}^{6}\right]\right\}$$

where *l*_0_ is 0.15300000 nm, *k_b_* is 186188.00 kJ/mol/nm^2^, *θ*_0_ is 113.600000°, and *k_θ_* is 488.272800 kJ/mol. *σ_ij_* is 0.358141284692 nm, and *ε_ij_* is 0.23430 kJ/mol.

The nanoparticle was initially placed 0.5 nm above one of the equilibrated DPPC monolayers, and the initial distance between the CNs and the DPPC monolayers was 0.7 nm (Figure 1b); this corresponded to the liquid condensed (LC) phase of the DPPC monolayers (the area per lipid was 0.5 nm^2^). A subsequent molecular dynamics simulation of 200 ns was performed after thousands of steps of energy minimization. The temperature was coupled to 310 K using the Nose–Hoover method, and a cutoff scheme of 1.2 nm was used for the non-bonded interactions. In addition, the Particle Mesh Ewald method with a fourier spacing of 0.1 nm was applied for the long-range electrostatic interactions [23]. All covalent bonds with hydrogen atoms were constraint using the LINCS algorithm [24].

The order parameter of the tail chain of the DPPC molecule represents the molecular orientation of the acyl chain, which can be calculated using Equation (3):

(3) $$S_{z,n}=\langle \frac{1}{2}\left(3\cos^{2}\theta_{n}\right)-1\rangle$$

where *θ_n_* represents the normal (Z-axis) angle between the vector connecting the *n* − 1 and *n* + 1 action groups of the DPPC tail chain and the DPPC monolayers. The order degree of the orientation of the DPPC molecules in different phases varies greatly. The greater the value of *S_z,n_*, the more ordered the orientation of the DPPC molecules [25].

The adsorption energy was calculated as the energy difference between the NP-DPPC system and their separate energies, and this was obtained using Equation (4) [26]:

(4) $$E_{\mathrm{ads}}=E_{\mathrm{NP}-\mathrm{DPPC}}-E_{\mathrm{NP}}-E_{\mathrm{DPPC}}$$

where *E*_NP_ and *E*_DPPC_ are the energies of NP and DPPC system, respectively. *E*_NP-DPPC_ is the energy of the whole system containing NP component and DPPC system.

## 3. Results and Discussion

### 3.1. Effect of CNs on the π-A Isotherms of the DPPC Monolayers

**Figure 2:**
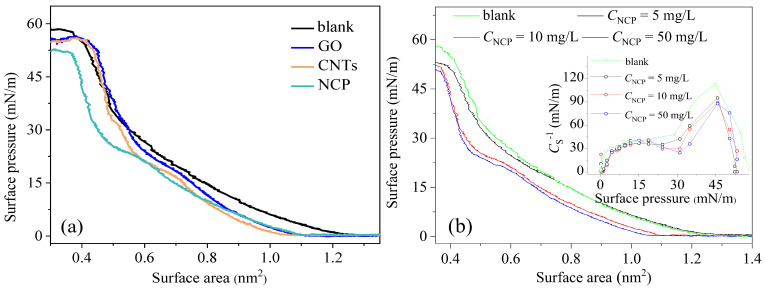
π-A isotherms of the DPPC monolayers over CNs subphases solution (*C*_CNs_ = 10 mg/L) (**a**) and π-A isotherms of the DPPC monolayers over NCP subphases solution (*C*_NCP_ = 0, 5, 10, 50 mg/L) (**b**); the inset shows the compressibility modulus values, *C*_S_^−1^, calculated from π-A isotherms for the DPPC monolayers with different NCP weight fraction.

The π-A isotherms of the phospholipid monolayers can be used to reflect the respiratory function [27]. The π-A isotherms of the DPPC monolayers in the presence of CNs are shown in Figure 2. The value of *π*_max_ for pure DPPC monolayer is approximately 59 mN/m at the temperature of 37 ± 0.5 °C. The phase coexistence plateau is unconspicuous. The principal behavior of phase transition and the relatively low value of *π*_max_ can be attributed to the high temperature. Higher temperature can make the monolayers become more fluid-like, and the values of π_max_ become smaller with increasing temperature [28,29]. The CNs exposure altered the shape of the DPPC isotherm and shifted the isotherm in relation to that corresponding to the pure DPPC. The π-A isotherms in the presence of CNs at a concentration of 10 mg/L were shifted to lower mean molecular areas compared to the isotherms of the pure DPPC monolayers (Figure 2a). When the layer was further compressed below a value of the area per molecule of about 0.6 nm^2^, the surface pressure reduced with a lower slope, and the phase coexistence plateau time increased in the presence of NCP. Upon further compression—in the presence of CNs and GO, but not of NCP—the isotherms overlapped with that of pure DPPC at surface pressures above 30–35 mN/m, which suggests a different retention of the nanomaterials upon compression. The observed isotherm overlap for DPPC with CNTs/GO (vs. NCP) may stem from two mechanisms: (1) CNTs/GO’s interfacial expulsion due to high aspect ratios and (2) nanocarbon powder’s irreversible lipid adsorption anchoring it at the interface. In addition, the maximum value of π obviously reduced in the presence of CNs, and this indicates that the compression resistance of the DPPC monolayers decreased. This effect was more pronounced for the DPPC monolayers in the presence of NCP. Hao et al. found that the adsorption of DPPC on magnetite nanoparticles can influence the phase behavior of DPPC [30]. Based on these statements, the forward shift of the compression isotherms implies the occurrence of the adsorption of the CNs to the DPPC. It is known that the surface area is an important indicator of the adsorption capacity of nanoparticles. However, the surface area of the three CNs was in the order of CNTs > NCP > GO (Table S1), which was inconsistent with the inhibition order (NCP > CNTs > GO). The stronger effect of NCP on the DPPC phase behavior was likely related to the hydrophilicity of the CNs that was in the order of NCP > CNTs > GO (Figure S4). Compared with NCP, the less-hydrophilic CNTs and GO may have incorporated into the DPPC monolayers, thereby increasing the surface density of the DPPC molecules to some degree [18].

Subsequently, NCP was used as a representative CN to further explore the influence of its concentration on the π-A isotherms of the DPPC monolayers. As shown in Figure 2b, the π-A isotherms of DPPC monolayers were shifted to a smaller area with an increasing concentration of NCP from 5 to 50 mg/L in the subphase. For pure DPPC, the behavior of the compressibility modulus values (*C*_S_^−1^ = −A(d_π_/d_A_)) against the surface pressure presents two maxima. The small maximum at low surface pressure corresponds to the elasticity of the liquid expansion (LE) phase, which is characterized by an intrinsic disorder. It is followed, during compression, by a minimum of elasticity due to the LE−liquid condensation (LC) coexistence phase. The second maximum is observed at high surface pressures and presents values (112 mN/m) of elasticity higher than those of the LE phase. This is attributed to the formation of an ordered LC phase. As shown in Figure 2b, the incorporation of NCP did not modify the qualitative feature of the elasticity. However, the maximum value of *C*_S_^−1^ was found to decrease with the incorporation of NCP, and the value was associated with the LE phase. It can be inferred that the strong hydrophobic cohesion typical of the condensed DPPC phase in pure monolayers is weakened by the presence of NCP, which is expected to disrupt their homogeneity.

All of these results together suggest that CN exposure can lead to an alternation of the phase behavior of the DPPC monolayers and weaken the elasticity of the DPPC monolayers. The results were similar to those found by Guzman et al. and showed that with an increase in hydrophobic nanoparticles in the DPPC monolayers, the interfacial film became more compressible [31,32]. The reduction in the elasticity of the DPPC monolayers may have been related to a lack of DPPC molecules in the air–water interface. The adsorption of CNs to the DPPC molecules influenced the directional arrangement of the DPPC molecules, and the random insertion of CNs probably induced phase segregation.

### 3.2. Effect of CNs on the π-t Curves of the DPPC Monolayers

**Figure 3:**
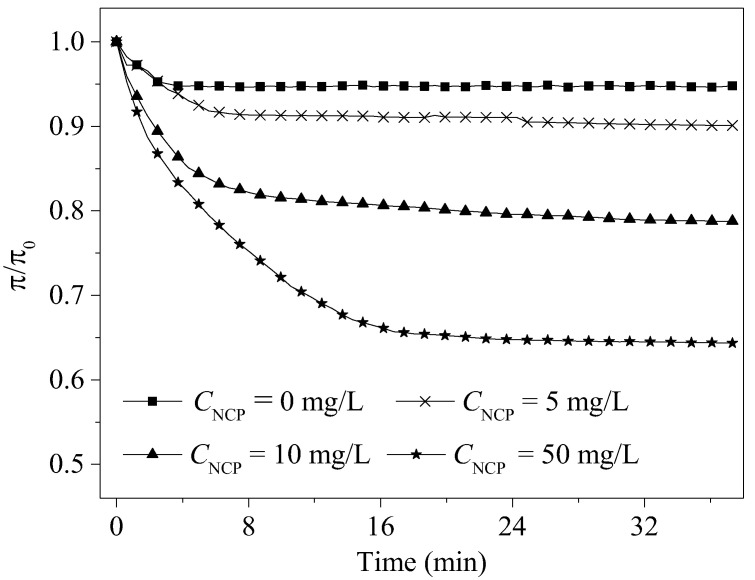
Normalization analysis of the π-t relaxation curves of the DPPC monolayers in the presence of NCP.

The relaxation process of the DPPC monolayers was studied using the π-t curves when the initial surface pressure was 30 mN/m. During the π-t curves test, the DPPC monolayers were in a constant compression area, and the variation curves of the surface pressure with time were recorded to study the relaxation process of the DPPC monolayers in the LE and LC phases in the presence of NCP. As shown in Figure 3, the steady-state surface pressure of the π-t curves of the pure DPPC monolayers was approximately 30 mN/m, and the π/π_0_ value was stable at approximately 0.95. When there was NCP in the subphase solution, the steady-state surface pressure of the π-t curves decreased gradually, and the surface pressure decreased gradually with an increase in NCP concentration in the subphase. This result showed that the higher the NCP concentration, the more DPPC molecules were adsorbed. This also explained the decrease in the surface pressure of the π-a isotherms in the air–water interface.

To further analyze the relaxation process of the DPPC monolayers in the presence of NCP, the π-t curves were fitted and analyzed using Equation (5):

π/π_0_ = C + ae^−t/τ^ (5)

where C represents the equilibrium pressure after curve normalization, and τ is the life cycle related to the DPPC monolayers self-assembly [33]. The fitting analysis results of the various parameters are shown in Table 1.

According to the fitting results in Table 1, with an increase in NCP concentration in the subphase solution, the C value decreased, that is, the surface pressure of the DPPC monolayers decreased when it reached equilibrium. This may have been due to the adsorption of the DPPC molecules by NCP. Under the adsorption, the DPPC molecules were closely bound to the surface of the NCP. This resulted in a decrease in the DPPC molecules that can normally have the function of surface activity at the interface and decrease the surface pressure of the monolayers. In addition, it was found that the value of τ increased at a high NCP concentration, and this indicated that the relaxation time of the DPPC monolayers became longer. This result indicates that the interfacial adsorption of the DPPC molecules by NCP may be an important reason why NCP affects the phase transition and compressibility of DPPC monolayers.

### 3.3. Morphology of the DPPC Monolayers Affected by CNs

**Figure 4:**
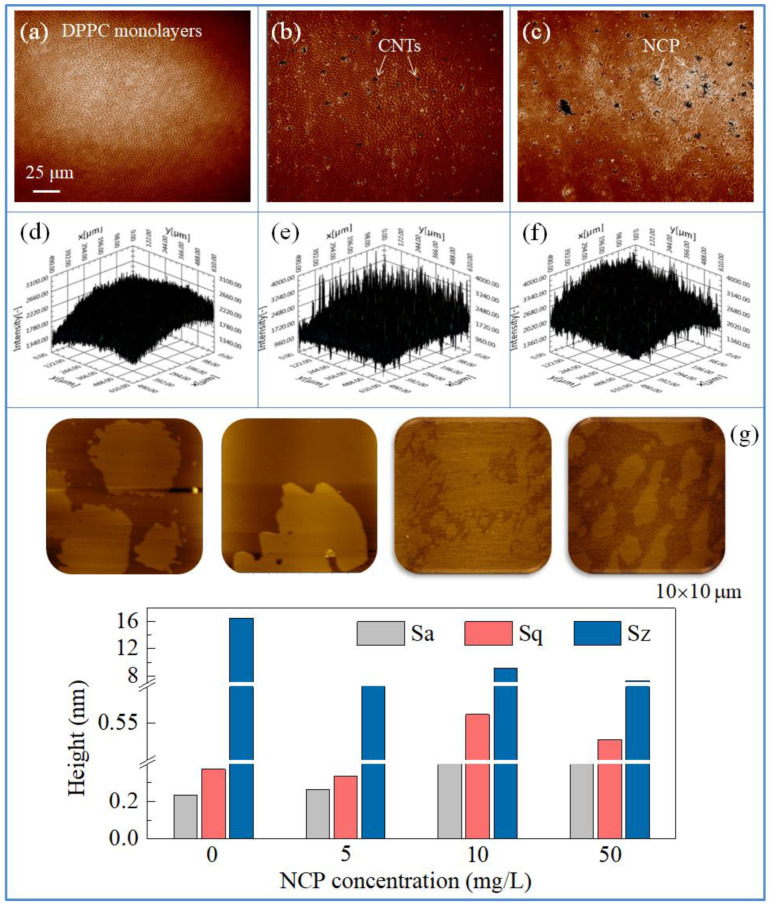
Effect of the CNs on the surface topography of the DPPC monolayers. (**a**–**f**): BAM images of pure DPPC monolayers and DPPC monolayers spread onto subphases with different CNs. (**a**): pure DPPC monolayers; (**b**): DPPC monolayers above CNTs solutions at a concentration of 10 mg/L; (**c**): DPPC monolayers above NCP solutions at a concentration of 10 mg/L; (**d**–**f**) show the intensity diagram corresponding to (**a**–**c**); (**g**): AFM characterization and height change of the DPPC monolayers above NCP solutions at the concentrations of 0, 5, 10, and 50 mg/L, respectively.

BAM is an effective method for investigating the lateral domains of lipid structures. The phase behavior and morphology of the DPPC monolayers are related to the surface pressure. During the respiratory cycle, the PS monolayers in the alveoli were subjected to a periodic area perturbation of about 30–40%, with a reference state characterized by a value of 30–40 mN/m. The experimental surface pressure was set to 30 mN/m, taking the values of surface pressure in biological films into account [34]. As shown in Figure 4a,d the lateral structure of the pure DPPC monolayers is uniformly flat and homogeneous, showing a typical LC phase, and this is consistent with previous reports [35]. In the presence of CNTs and NCP, the DPPC monolayers existed in the LE phase, and the ordered structure of the monolayers was destroyed by the CNs (Figure 4b,c). At the same concentration, the disturbance of NCP to the DPPC monolayers was more obvious than that of the CNTs, which was consistent with the π-A isotherms. Figure 4d,f represent the reflection strengths of the DPPC monolayers at the air–water interface. It was found that the reflection intensity fluctuated greatly in the presence of CNs, which confirmed the strong disturbance of CNs to the DPPC monolayers.

AFM was employed to further investigate the effect of CNs on the morphology of the DPPC monolayers. As shown in Figure 4g, the surface of the pure DPPC monolayers was relatively flat, and with the addition of NCP in the subphase solution, the topographic images showed many holes in the DPPC monolayers. In addition, the bulk density of the DPPC molecules decreased. Compared with the pure DPPC monolayers, the roughness of the monolayers over the NCP aqueous subphase significantly increased. The arithmetical mean height (Sa) increased from 0.232 nm to 0.263, 0.445, and 0.429 nm, and the root mean square height (Sq) increased from 0.373 nm to 0.336, 0.562, and 0.528 nm. This topographic change corresponded to the monolayer phase transition. As there was a difference in the respective thickness values of the monolayers in both the LE and LC phases, the coexistence of LE and LC phases could result in a sharp increase in roughness.

This result further indicates that the incorporation of CNs into DPPC monolayers will affect the LC phase formation of the DPPC monolayers. Nanoparticles can interfere with the ultrastructure of DPPC monolayers. However, the phase transition of DPPC monolayers is the key factor for the PS film to reduce the surface tension of the air–water interface. Previous studies have confirmed that phospholipid molecules in the PS film will undergo a periodic phase transition during the compression expansion cycle, and DPPC molecules will change from the “fluid” LE phase to the “solid” LC phase. This phase transition and phase separation play important roles in regulating the biophysical function of the PS film [36,37]. Therefore, the effect of CNs on the ultrastructure of the DPPC monolayers may further lead to physiological dysfunction of the PS film.

### 3.4. Disturbance of CNs on the Structure of the DPPC Monolayers

**Figure 5:**
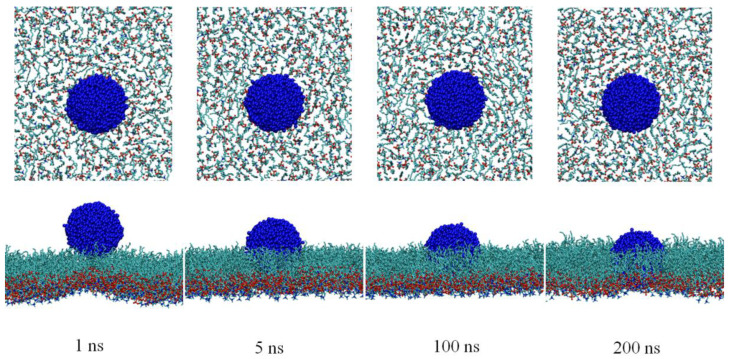
Structural perturbation of the DPPC monolayers affected by CNs.

To reveal the interactions between CNs and the DPPC monolayers at the molecular level, the particle translocation through the DPPC monolayers was simulated. Because the interaction between CNs and the PS film is related to the phase transition of the PS film, a CN was initially contacted to the DPPC monolayers during the LC phase from the air phase to mimic the cases of inhaled CNs. Representative snapshots depicting the side views of the CNs interacting with the DPPC monolayers are presented in Figure 5. It was found that the hydrophobic CN could easily spread on the DPPC monolayers, and the contact area increased significantly. During the entire process of contact, the CN became tightly immersed in the hydrophobic region of the DPPC monolayers and encountered increased difficulties in crossing the DPPC monolayers. In addition, they may have even disrupted the structure of the DPPC monolayers. At a spatial scale of 200 ns, the transmembrane behavior of the CN was not observed.

**Figure 6:**
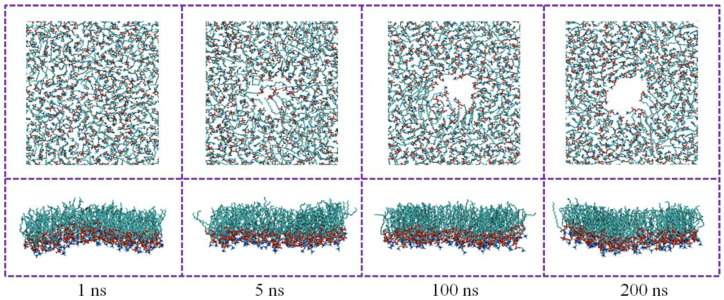
Characterization of the DPPC monolayers perturbation by deposited CN. (Time sequence of typical snapshots from the top view. The carbon nanoparticle was not shown for clarity).

As shown in Figure 6, after the CN with a particle size of 3.0 nm contacted the DPPC monolayers, the hydrophobic acyl chain region of the DPPC monolayers was occupied, while the hydrophilic head was less affected by the particle. With increasing time, the pore structure induced by the CN became larger. For the DPPC monolayers in the LC phase, under the disturbance of the CN, the DPPC monolayers changed from a uniform and continuous matrix structure to a disordered structure with phase state separation.

### 3.5. Effect of CNs on the Order Degree of the DPPC Monolayers

**Figure 7:**
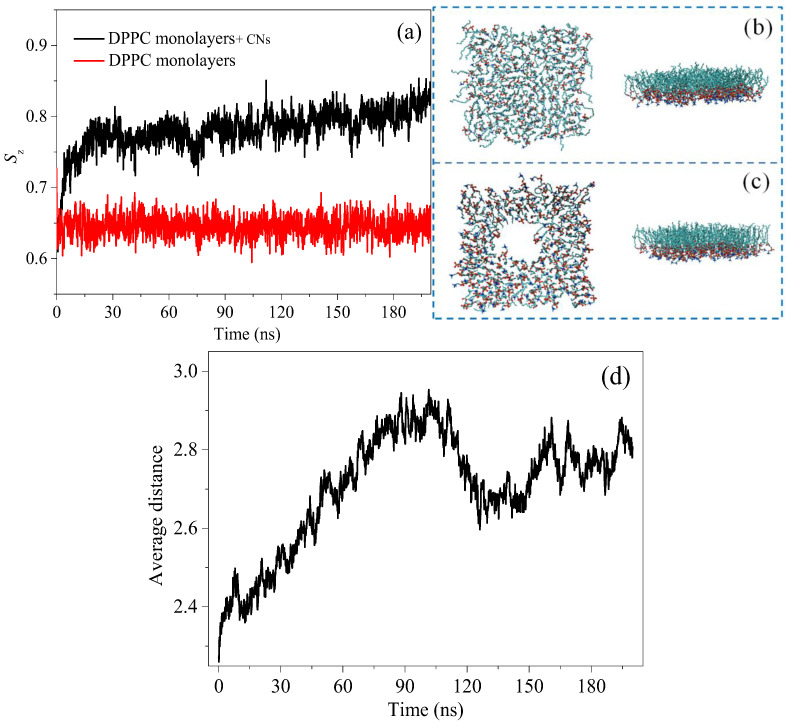
Influence of CNs on the ordering degree of the DPPC monolayers. (**a**): changes to ordering parameters of DPPC monolayers; (**b**): structure of DPPC monolayers; (**c**): changes to DPPC monolayers structure caused by CNs disturbance; (**d**): the average distance between P atoms in DPPC monolayers with simulation time.

To study the change in the order degree of the DPPC monolayers affected by CNs, the order parameter, *S*_z_, was used to analyze the change law of the order parameters of the DPPC molecules. *S*_z_ can describe the inclination, chain orientation, and translation periodicity of molecules. The larger its value is, the smaller the angle between the hydrophobic acyl chain of phospholipid molecules and the z-axis is, and the better the order is [25,38]. As shown in Figure 7a, the DPPC monolayers are closely arranged at the air–water interface, and the order parameters of the tail chains of the DPPC molecules are large, which corresponds to the LC state. Interestingly, the value of *S*_z_ increased during the contact between the CNs and the DPPC monolayers. Although this result seems to contradict the destruction of the monolayers, in fact, after the addition of CNs, the DPPC molecules far away from the CNs were squeezed to varying degrees. Therefore, the increase in the order parameters may have been caused by extrusion between the distal DPPC molecules. In addition, an increase in the order parameters of the DPPC molecules will increase the monolayers thickness, and this also explains the height change of the DPPC monolayers in the AFM. To further investigate the effect of CNs on the bulk density of the DPPC monolayers, the average distance between the P atoms in the DPPC monolayer was analyzed, as shown in Figure 7b–d. It was observed that the distance between the DPPC molecules increase was affected by the CNs at the spatial scale of 200 ns. Interestingly, the average distance between the P atoms fluctuated, as shown in Figure 7d. The binding affinity of the CNs with the DPPC molecules induced the DPPC monolayers to form a pore structure; thus, the average distance between P atoms in DPPC monolayer increased. However, with particle incorporation into the DPPC monolayers, the bulk density of the DPPC molecules was slightly increased under the extrusion of particles. Thus, during the entire process, the adsorption and extrusion of the CN and the DPPC molecules occurred simultaneously, which caused a fluctuation in the distance between P atoms. This result indicated that the deposition of CNs on the DPPC monolayer was accompanied by the adsorption and extrusion of DPPC molecules, and adsorption was dominant. The incorporation of CNs modifies the packing of the DPPC molecules at the interface and their lateral cohesion, worsening the mechanical performance of the model PS film. This may induce a modification of the normal physiological function of the PS film.

### 3.6. Adsorption of the DPPC Molecules by CNs

**Figure 8:**
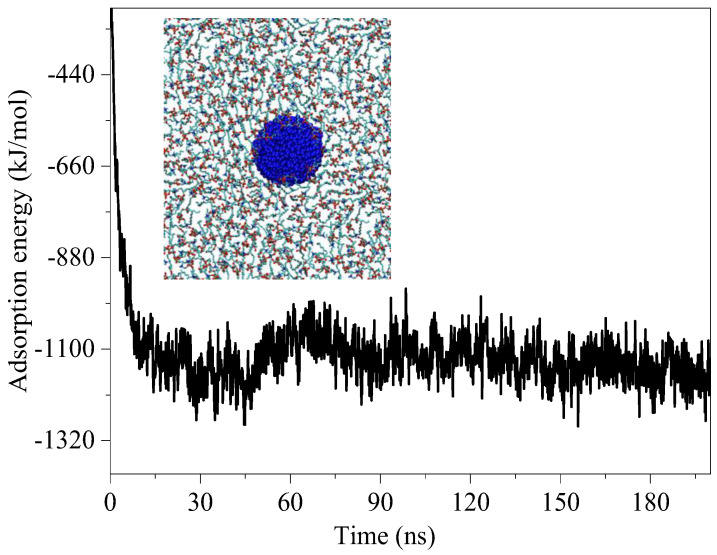
Adsorption energy of CN for the DPPC molecules.

To investigate the binding ability of CNs to the DPPC molecules, the adsorption energy of the CNs to the DPPC molecules was calculated. As shown in Figure 8, the energy of the system was relatively high at the beginning of the simulation, and then the system energy tended to be stable and reached a stable state. According to Equation (4), the average adsorption energy of the CNs and DPPC molecules was approximately 1100 kJ/mol, which is the characteristic of chemical adsorption energy. This result indicates that the CNs had strong binding affinities with the DPPC molecules, and this caused the CNs to spontaneously adsorb the DPPC molecules. The adsorption of CNs to the DPPC molecules will induce the monolayers to form a pore structure. It has been reported that DPPC is primarily responsible for the low surface tension of the PS film [19,20]. A relative surfactant deficiency and dysfunction can cause disease states such as respiratory distress syndrome [39]. Hence, the surfactant inhibition induced by CNs can indeed be physiologically relevant.

Previous studies have shown that when nanoparticles adsorb onto the DPPC monolayers, they change the local properties of lipids [40]. This may initiate the structural disruption of lipid monolayers. Schleh and Hohlfeld pointed out that the particles deposited on the PS film will be surrounded by the hydrophobic tails of phospholipids to form vesicles [41]. Many studies have shown that phospholipid vesicles on the surface of particles can significantly affect the dispersion of particles. In our previous studies, it was found that DPPC caused the agglomeration of NCP and GO [42]. Kendall and Zhou et al. also found that DPPC in simulated lung fluid led to the agglomeration of fine particles [43,44]. It can be inferred that the adsorption of CNs to DPPC molecules may not only disturb the interfacial structure of the PS film, but also, the migration and transport of particles will be changed.

## 4. Conclusions

The interaction between CNs and the PS film based on changes in the interfacial properties of the DPPC monolayers was successfully investigated in this study. Based on the results, it can be concluded that CN exposure can hinder the normal phase transition behavior of DPPC monolayers, inhibit the ability of the DPPC monolayers to reduce surface tension, and reduce the stability of DPPC monolayers during the compression of DPPC molecules. The AFM images showed that the surface topography of the DPPC monolayers was destroyed as a result of CNs disturbance. The MD simulation demonstrated that there was a strong binding trend between the DPPC molecules and the CNs. The DPPC molecules were spontaneously adsorbed to the surface of CNs. The orientation of the DPPC molecules close to the CNs was disordered, and the packing density of the DPPC molecules was reduced. This study confirmed that CNs changed the relaxation mechanism of the DPPC monolayers, and the adsorption between the DPPC and the CNs was an important mechanism of the change in the DPPC interfacial properties. Our data clearly showed that CNs can alter the phase behavior and microstructure of the PS film. In particular, this study revealed that the adsorption of lipids onto surfaces of CNs was the major mechanism through which CNs affected the physicochemical function of the PS film. The CNs-DPPC interaction mechanism provided evidence for the inhibitory effect of CNs on the PS film at the molecular level. In addition to the specific results, this study adopted combined laboratory experiments and MD simulations to evaluate the mechanical response of the PS film affected by CNs. These interesting working elements can be considered promising for in vitro toxicology assays of nanoparticles on PS film, and the results help deepen our understanding of the toxic effect of CNs on lung at the molecular level.

## Figures and Tables

**Table 1:** Parameters obtained from π-t curve fitting analysis (π = 30 mN/m).

*C*_NCP_ (mg/L)	C	a	τ	r^2^
0	0.948	0.054	1.284	0.977
5	0.902	0.090	4.351	0.929
10	0.788	0.192	4.717	0.965
50	0.643	0.346	6.363	0.995

## Data Availability

The data that support the findings of this study are available upon request from the authors.

## Supplementary Materials

The following supporting information can be downloaded at: https://www.mdpi.com/article/10.3390/nano15161244/s1, Text S1: the hydrophilicity/hydrophobicity of the CNs; Table S1: properties of NCP, CNTs and GO; Figure S1: typical SEM images of NCP, CNTs and GO; Figure S2: size distribution and hydrodynamic diameters of NCP, CNTs and GO at the concentration of 50 mg/L in pure water; Figure S3: zeta potentials of NCP, CNTs and GO at the concentration of 50 mg/L in pure water; Figure S4: a direct visual demonstration of the hydrophilicity/hydrophobicity of NCP, CNTs and GO; and Figure S5: construction of the spherical amorphous CN.

## Abbreviations

The following abbreviations are used in this manuscript:

CNs	Carbon nanoparticles
DPPC	1,2-dipalmitoyl-sn-glycero-3-phosphocholine
PS	Pulmonary surfactant
NCP	Nanocarbon powder
GO	Graphene oxide
CNTs	Carbon nanotubes
BAM	Brewster angle microscope
AFM	Atomic force microscope
LE	Liquid expansion
LC	Liquid condensation
