# Supplementary material for: Evaluating the Impact of Carbon Nanoparticles on the Interfacial Properties of the Pulmonary Surfactant Film
Source: Nanomaterials (Basel). 2025 Aug 14;15(16):1244. doi: 10.3390/nano15161244 (PMC12388053; doi:10.3390/nano15161244)
Supplement: Supplementary file 1 [file nanomaterials-15-01244-s001.zip › nanomaterials-3774097-supplementary.pdf]

nanomaterials

Supplementary Materials

Evaluating the Impact of Carbon Nanoparticles on the Interfacial Properties of the Pulmonary Surfactant Film

Yingxue Geng [1,2], Qun Zhao [2,*], Junfeng Wang [1], Yan Cao [2], Yunshan Wang [1], Wenshi Gou [1], Linfeng Zhang [2] and Senlin Tian [2,*]

[1] Faculty of Civil and Hydraulic Engineering, Xichang University, Xichang 615013, China; xcc04100056@xcc.edu.cn (Y.G.); xcc04100057@xcc.edu.cn (J.W.); xcc03000038@xcc.edu.cn (Y.W.); xcxygws@xcc.edu.cn (W.G.)
[2] Faculty of Environmental Science and Engineering, Kunming University of Science and Technology, Kunming 650500, China; 20250072@kust.edu.cn (Y.C.); zhanglinfeng@stu.kust.edu.cn (L.Z.)
* Correspondence: 20190073@kust.edu.cn (Q.Z.); 20050149@kust.edu.cn (S.T.)

**Text S1**	**The hydrophilicity/hydrophobicity of the CNs**
Table S1	Properties of NCP, CNTs and GO
Figure S1	Typical SEM images of NCP (a), CNTs (b) and GO (c)
Figure S2	Size distribution and hydrodynamic diameters of NCP (a), CNTs (b) and GO (c) at the concentration of 50 mg/L in pure water
Figure S3	Zeta potentials of NCP, CNTs and GO at the concentration of 50 mg/L in pure water
Figure S4	A direct visual demonstration of the hydrophilicity/hydrophobicity of NCP (a), CNTs (b) and GO (c)
Figure S5	Construction of the spherical amorphous CN

Academic Editor(s): David B. Alexander

Received: 7 July 2025
Revised: 3 August 2025
Accepted: 8 August 2025
Published: date

Citation: Geng, Y.; Zhao, Q.; Wang, J.; Cao, Y.; Wang, Y.; Gou, W.; Zhang, L.; Tian, S. Evaluating the Impact of Carbon Nanoparticles on the Interfacial Properties of the Pulmonary Surfactant Film. *Nanomaterials* **2025**, *15*, x. https://doi.org/10.3390/xxxxx

Copyright: © 2025 by the authors. Submitted for possible open access publication under the terms and conditions of the Creative Commons Attribution (CC BY) license (https://creativecommons.org/licenses/by/4.0/).

Text S1 The hydrophilicity/hydrophobicity of the CNs.

Figure S4 shows a direct visual demonstration of the hydrophilicity/hydrophobicity of the CNs. The contact angles of the three CNs are less than 90°, indicating that they are all hydrophilic. The order of contact angles was as follows: NCP < CNTs < GO, which means that the hydrophilicity of the three CNs was in the order of NCP > CNTs > GO.

Table S1. Properties of NCP, CNTs and GO.

Properties	CNs		
	NCP	CNTs	GO
Diameter (nm)	30	20–40	500–5000
Thickness (nm)	-	-	0.8–1.2
Single layer ratio (%)	-	-	~99
Surface area (m^2/g)	21.96 ± 1.64	70–150	1.50 ± 0.06
Pore diameter (nm)	3.40 ± 0.012	32.25 ± 0.48	3.41 ± 0.022

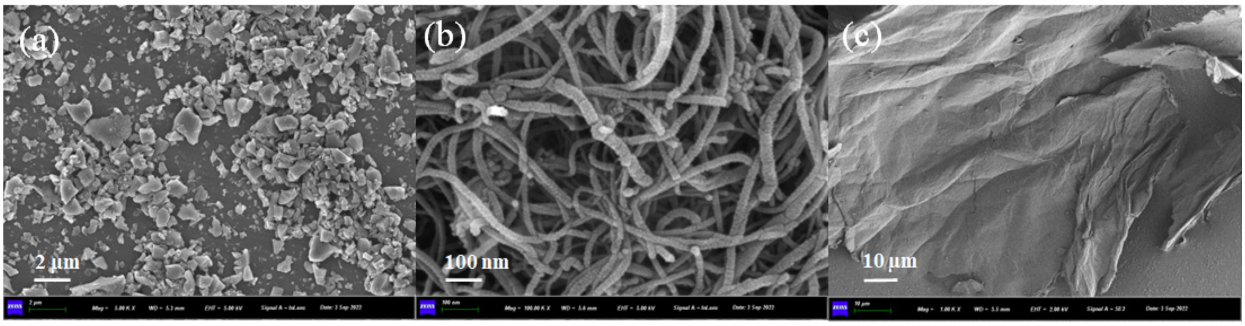

Figure S1. Typical SEM images of NCP (a), CNTs (b) and GO (c).

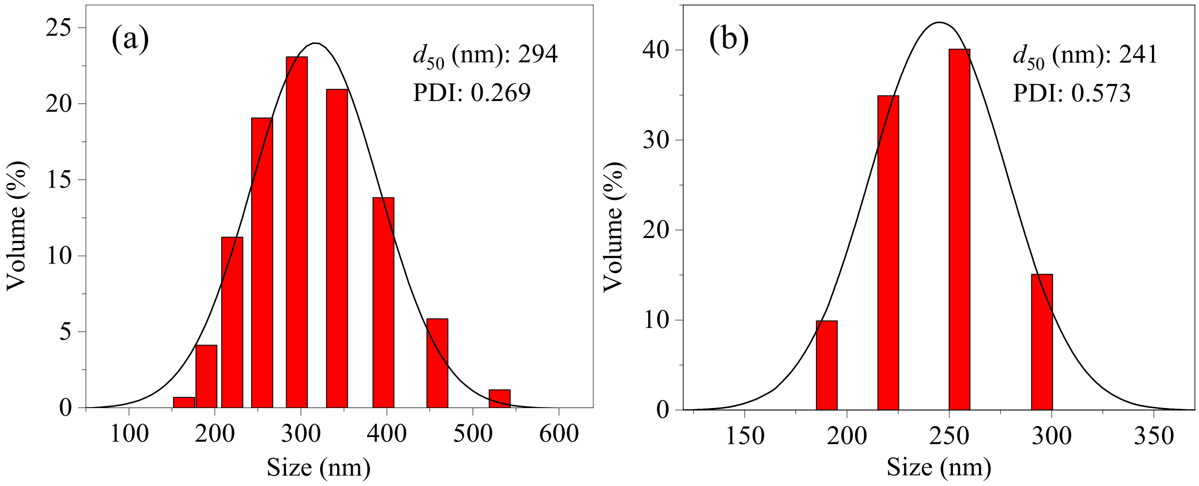

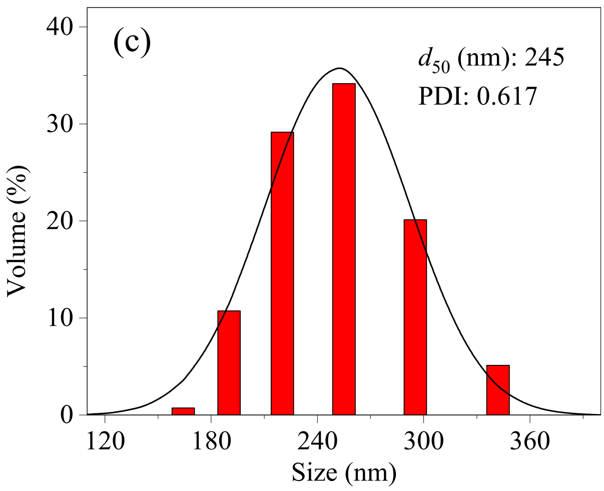

Figure S2. Size distribution and hydrodynamic diameters of NCP (a), CNTs (b) and GO (c) at the concentration of 50 mg/L in pure water.

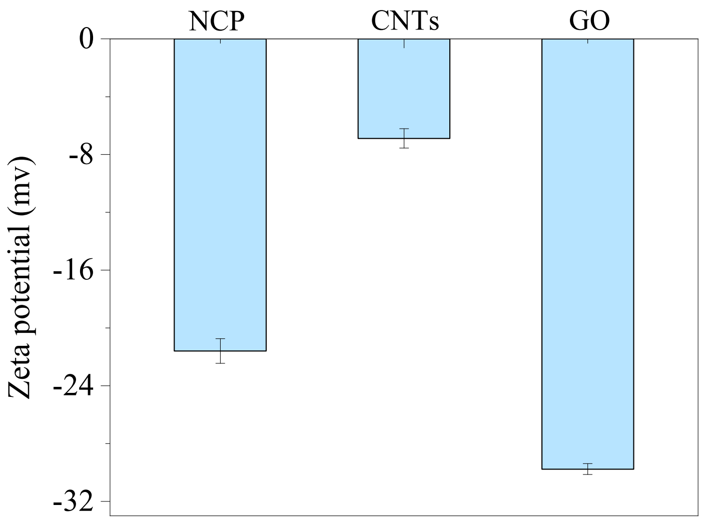

Figure S3. Zeta potentials of NCP, CNTs and GO at the concentration of 50 mg/L in pure water.

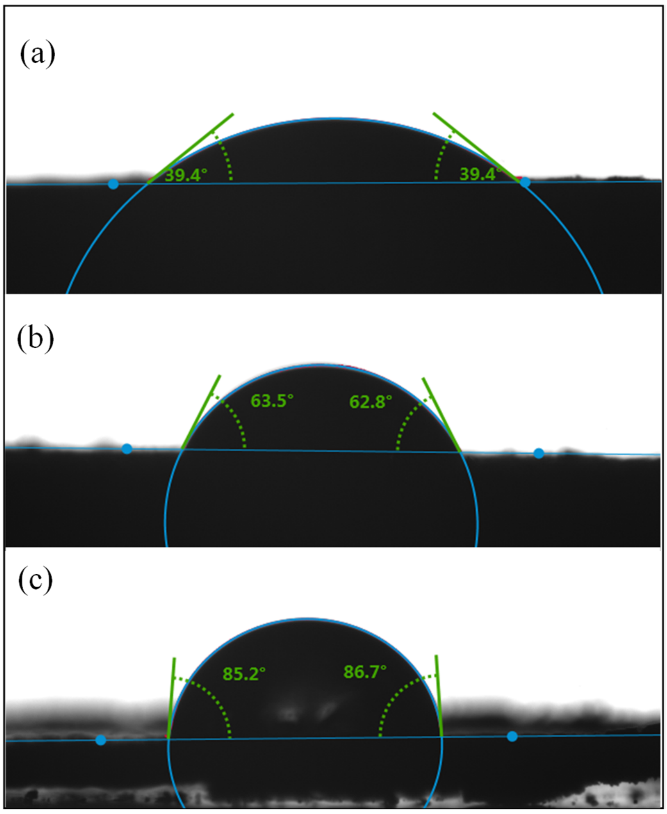

Figure S4. A direct visual demonstration of the hydrophilicity/hydrophobicity of NCP (a), CNTs (b) and GO (c).

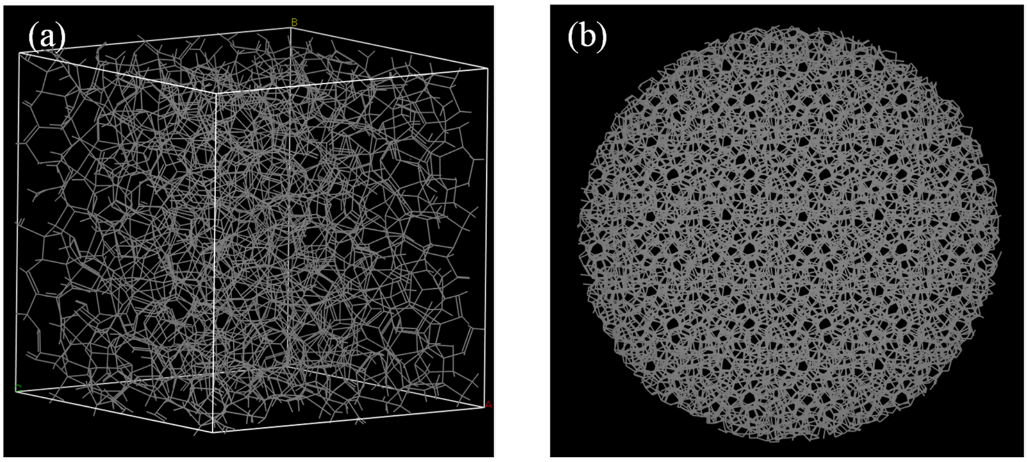

Figure S5. Construction of the spherical amorphous CN (a: amorphous carbon periodic structure, b: spherical amorphous carbon system).
